# Melatonin enhanced the cardioprotective effects of HTK solution on Langendorff-perfused mouse hearts subjected to ischemia/reperfusion

**DOI:** 10.22038/IJBMS.2023.74152.16109

**Published:** 2024

**Authors:** Mingchu Sun, Zihui Zhang, Yue Yin, Lu Yu, Wenhua Jiang, Chan Zhang, Chunhu Gu, Heng Ma, Yishi Wang

**Affiliations:** 1 Institute of Medical Research, Northwestern Polytechnical University, Xi’an Shaanxi, 710072 China; 2 Department of Physiology and Pathophysiology, School of Basic Medicine, Fourth Military Medical University, Xi’an, 710032 China; 3 Department of Pathology, Xijing Hospital, Fourth Military Medical University, Xi’an 710032, China; 4 Department of Cardiovascular Surgery, Xijing Hospital, Fourth Military Medical University, Xi’an 710032, China

**Keywords:** Endoplasmic reticulum-stress, Heart protection, Langendorff heart, Melatonin, Myocardial, ischemia/reperfusion injury

## Abstract

**Objective(s):**

Cardiac arrest is a crucial procedure in various cardiac surgeries, during which the heart is subjected to an ischemic state. The occurrence of ischemia/reperfusion (I/R) injury is inevitable due to aortic blockage and opening. The Histidine-tryptophan-ketoglutarate (HTK) solution is commonly used as an organ protection liquid to mitigate cardiac injury during cardiac surgery. Despite its widespread use, there is significant potential for improving its protective efficacy.

**Materials and Methods:**

The cardioprotective effect of HTK solution with and without melatonin was evaluated using the isolated Langendorff-perfused mouse heart model. The isolated C57bL/6 mouse hearts were randomly divided into four groups: control, I/R, HTK solution treatment before reperfusion (HTK+I/R), and HTK solution combined with melatonin before reperfusion (HTK+M+I/R). Cardiac function and myocardial injury markers were then measured. AMP-activated protein kinase α2 (AMPKα2) KO mice were used to investigate the underlying mechanism.

**Results:**

In our study, we found that melatonin significantly improved the protective effects of HTK solution in an isolated Langendorff-perfused mouse model, mechanistically by reducing mitochondrial damage, improving energy metabolism, inhibiting cardiomyocyte apoptosis, and reducing myocardial infarction size. We also observed that the HTK solution alone was ineffective in inhibiting ER stress, but when melatonin was added, there was a significant reduction in ER stress. Furthermore, melatonin was found to alleviate carbonyl stress during cardiac I/R. Interestingly, our results showed that the cardioprotective properties of melatonin were dependent on AMPKα2.

**Conclusion:**

The findings presented in this study offer a valuable empirical foundation for the development of perioperative cardioprotective strategies.

## Introduction

The HTK solution was initially developed by Brestschneider *et al*. in 1975 for the preservation of cardiac arrest patients and has since been widely used in clinical heart transplantation. It is characterized by a robust histidine-based buffer system with low sodium and slightly elevated potassium levels. This solution not only provides myocardial protection in cardiac surgery but also protects of transplanted organs such as the heart, kidney, liver, and pancreas (1). Numerous studies have demonstrated that HTK is a relatively safe and effective alternative to multidose cardioplegias for myocardial protection during surgical correction of acquired pathology. It is one of the most commonly used cardiac preservation solutions to reduce ischemia/reperfusion (I/R) injury during surgery (2, 3). However, there is still significant room for improvement in understanding the protective effects of HTK solution on I/R injury. In recent years, there has been a continuous emergence of studies focusing on the enhancement of HTK solutions. Several studies have shown that the addition of compound glycyrrhizin to HTK solution can mitigate cold ischemic injury during cold storage (4). Furthermore, exposure to HTK solution has been found to induce ROS, but when co-administered with 8-hydroxy-2’-deoxyguanosine, it attenuates ROS-mediated sodium bicarbonate cotransporter activity, reduces ROS levels, and decreases the expression of apoptotic markers and fibrosis-associated proteins in cardiac cells (5). Histidine has been shown to accelerate the formation of ROS (6), and it has been reported that N-acetylhistidine is used as a substitute for a portion of histidine (7). While HTK is the most commonly used and effective cardioplegic solution in clinical practice, finding effective strategies to enhance its effects is of great value in inhibiting I/R injury in cardiac surgery.

Melatonin, an amine hormone produced by the pineal gland, has been proven to possess powerful antioxidant properties (8). It acts by scavenging free radicals, preventing oxidation, and inhibiting lipid peroxidation, which in turn protects cell structure, prevents DNA damage, and reduces the content of peroxides (9). Melatonin exhibits strong antioxidant and anti-inflammatory effects and it plays protective roles in various cardiovascular diseases, including hypertension, ischemic heart disease, and atherosclerosis. Recent studies have demonstrated that melatonin in combination with glucosamine, can effectively protect kidney tissue for up to 72 hr (10). However, the potential of melatonin to enhance the myocardial protective effect of HTK solution and the underlying mechanism remains unknown. 

The purpose of this investigation was to examine the potential of melatonin in enhancing the cardioprotective properties of HTK solution for preventing I/R injury. Additionally, this study aimed to understand the underlying mechanisms involved.

## Materials and Methods

***Animals***

Adult male C57BL/6 mice (3-4 months) were purchased from the Animal Experimental Center of Air Force Military Medical University. AMPKα2 knockout mice were purchased from the Animal Center of Jackson Laboratory. All experimental animals were housed under specific pathogen-free (SPF), constant temperature (25±2 ^°^C), and constant humidity (45%-50%), and maintained a circadian rhythm for 12 hr. All animal experiments are approved by the Ethics Committee of the Air Force military Medical University and meet the requirements of laboratory animal ethics and welfare.

***Reagents***

The citrate synthase assay kit and ATP synthase activity assay kit were from GENMED Scientific Inc. (Arlington, MA, USA). Sodium azide (NaN_3_) was purchased from Flash Molecular Biotechnology (Shanghai, China). Bovine serum albumin (BSA) and methanol were from Kehao Biological Engineering (Xi’an, China). Cell lysate (RIPA), ECL luminescent solution, primary antibody diluent, and PBS were from Beyotime (Nantong, China). Protease inhibitor, Tubulin monoclonal antibody, Carbonyl cyanide-m-chlorophenylhydrazone (CCCP), Dimethyl sulfoxide (DMSO), and 2,3,5-triphenyltetrazolium (TTC) were obtained from Sigma-Aldrich (St. Louis, MO, USA). The antibody removal solution was from Nanjing Jiancheng Bioengineering Institute (Nanjing, China). Horse radish peroxidase (HRP) labeled secondary antibody was from Millipore Company (Millipore, USA). Antibodies to acetaldehyde dehydrogenase 2 (ALDH2), p-AMPK, AMPKα1, tubulin, Protein kinase R-like ER kinase (PERK), p-PERK, eukaryotic translation initiation factor 2 α (eIF2α), p-eIF2α, activating transcription factor 4 (ATF4), and C/EBP-homologous protein (CHOP) were from Cell Signaling Technology (Danvers, MA, USA).

***Experimental design and Langendorff-perfusion of hearts***

Mice were anesthetized by intraperitoneal injection of pentobarbital sodium (40 mg/kg). After anesthesia, the heart was quickly removed and installed on a Langendorff heart perfusion device for aortic perfusion (temperature 36.5±0.5 ^°^C, pH 7.4±0.02). Isolated hearts were randomly divided into four groups: (I) control, (II) I/R, (III) HTK solution treatment before reperfusion (HTK+I/R), and (IV) HTK solution in combination with melatonin before reperfusion (Mel+HTK+I/R). 

The hearts were quickly excised and retrogradely perfused (4 ml/min) on a Heart Perfusion System (Radnoti Glass Technology, Monrovia, CA, USA) with 95% O_2 _and 5% CO_2_ equilibrated Krebs-Henseleit buffer (118 mM NaCl, 4.75 mM KCl, 1.2 mM KH_2_PO_4_, 1.2 mM MgSO_4_, 25 mM NaHCO_3_, 1.4 mM CaCl_2_) containing 7 mmol/l glucose, 0.4 mmol/l oleate, 1% BSA, and a low fasting concentration of insulin (10 µU/ml)(11). The heart was perfused for 10 min to achieve hemodynamic balance. 

Subsequently, the mouse heart was subjected to continuous perfusion at a temperature of 4 ^°^C, with a constant flow rate of HTK solution at a pressure of 10 cm water column for a duration of 10 min. To achieve a concentration of 50 μmol/l, melatonin was initially dissolved in 1.5 ml ethanol and subsequently added to HTK (12).

For generating the *ex vivo *ischemic model, buffer flow was cut off for 30 min after reperfusion of HTK solution with and without melatonin. The hearts were then reperfused with the same rate of Krebs-Henseleit buffer flow during reperfusion. The LabChart6 software (AD Instruments) was used to monitor the heart rate and left ventricular developed pressure, as described previously (13). Cardiac function, cTnl, and LDH levels were measured every 10 min for 30 min during the reperfusion period, and myocardial infarct size was measured after 120 min of reperfusion.

***Cardiac function measurement***

During the reperfusion period, a liquid-filled latex balloon connected to a solid pressure sensor was protruded into the left ventricle by the left atriotomy to measure pressure. The heart rate left ventricular diastolic pressure (LVDP) and maximum and minimum pressure change rate (dP/dtmax, dP/dtmin) were recorded by a digital acquisition system.

***Measurement of myocardial infarct size***

TTC was dissolved in PBS buffer solution at a concentration of 0.5%. The isolated heart after 120 min of ischemia and reperfusion was injected into the aorta 5 times at 37 ^°^C, each time 0.2-0.4 ml for 5 min, then the heart was stored in a -20 ^°^C refrigerator for 30 min, and then removed. The heart was cut into 5 myocardial slices perpendicular to the long axis from the apex to the bottom of the heart. Fixed with 10% formaldehyde for 1-2 hr, photographed with a Lycra microscope, and analyzed with the NIH image software.

***Determination of lactate dehydrogenase (LDH) and troponin***

Coronary artery outflow samples were collected from isolated hearts during I/R. LDH and cardiac troponin I (cTnl) levels were measured by LDH and cTnl kits (Sigma, USA), respectively. The effluent samples were collected every 10 min from the reperfusion period to the end of reperfusion and stored in a refrigerator at -80 ^°^C for spectrophotometer analysis.

***Determination of the activity of ATP synthetase and citrate synthetase***

Myocardial citrate synthase activity and ATP synthase activity were determined according to the instructions of citrate synthase and ATP synthase activity determination kit (GENMED Scientific Inc.).

***Myocardial mitochondrial morphology by electron microscope***

The heart tissue was cut into cubes (1mm×1mm×1mm) and fixed with 2.5% glutaraldehyde and 1% osmium tetroxide (pH=7.2). The monolayer was embedded in LX-112 (Ladd research, Burlington, VT, USA) and stained with uranyl acetate and lead citrate. The sections were magnified and observed by electron microscope. The size, shape, and number of mitochondria were analyzed by Image-Pro Plus software.

***Determination of ALDH2 activity***

Fifty micrograms of protein were extracted and placed in sodium pyrophosphate (33 mmol/l) containing 15 μmol/l propionaldehyde and 0.8 mmol/l NAD^+^. The activity of ALDH2 was quantitatively determined by colorimetry. Briefly, propanol, the substrate of ALDH2, is oxidized in propionic acid, and NAD^+^ is degraded by NADH to measure the activity of ALDH2. In the process of converting NAD^+^ to NADH, the absorbance of 340 nm was detected by an ultraviolet spectrophotometer to convert the enzyme activity of ALDH2. The extinction coefficient of NADH at 340 nm is 6.22 mmol. The enzyme activity of ALDH2 was expressed by NADH (min mg protein).

***Western blotting***

About 100 mg heart tissue was homogenized 4-5 times with Diax 900 homogenizer for 10 sec each time and then centrifuged at 4 ^°^C for 20 min at 12000 rpm. The protein concentration was quantified according to the BCA protein quantitative kit (Pierce Company, USA). Twenty micrograms of protein is added for SDS-PAGE. After electrophoresis, the gel was transferred to the PVDF membrane. Then the membrane was sealed at 5% BSA for 1 hr at room temperature, and incubated with the antibodies to PERK (1:1000), p-PERK (1:1000), eIF2α (1:1000), p-eIF2α (1:1000), ATF4 (1:1000), CHOP (1:1000), AMPKα2 (1:1000), p-AMPK (Thr172)(1:1000), and tubulin (1:1000) at 4 ^°^C overnight. After washing the membrane with PBST, the membranes were incubated with HRP-labeled antibodies for 1 hr at room temperature. ECL luminescent solution was used to determine the protein expression using a Bio-Rad gel imaging system.

***Statistical analysis***

The data were statistically analyzed by GraphPad Prism 9.0 software (GraphPad Software, San Diego, CA, USA), and the experimental data were expressed in mean±standard error. T-test was used to compare the data between the two groups, and single factor analysis of variance (one-way analysis variance, one-way ANOVA) was used to compare the data for more than two groups with the SNK-q test to further analyze the significant differences between the two groups. *P*<0.05 was considered statistically significant.

## Results

***HTK solution alleviated I/R injury in isolated heart***

**Figure 1:**
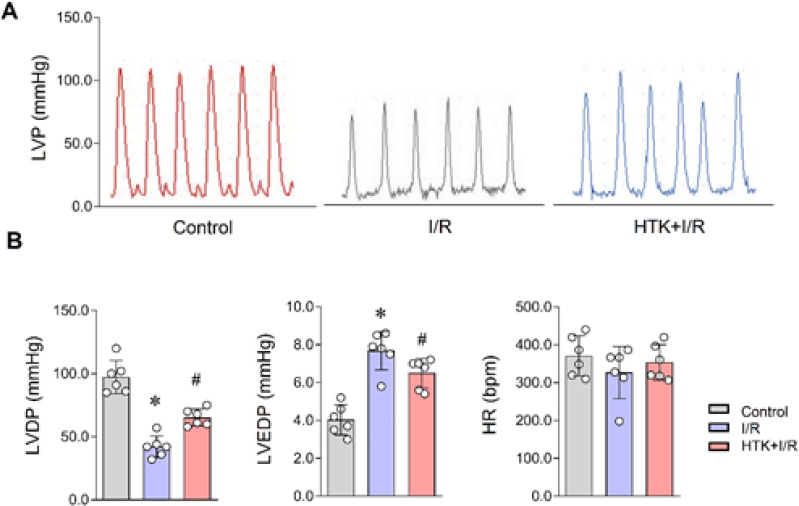
HTK solution served beneficial effects on cardiac function in isolate perfused mice heart

To investigate the protective effects of HTK solution on I/R injury, we first perfused the isolated hearts of mice and then randomly divided them into three groups: control, I/R, and HTK+I/R. The findings revealed that compared to the control group, incubation with HTK solution partially mitigated the myocardial injury caused by reperfusion after cardiac arrest and partially restored the left ventricular pressure (LVP)(Figure 1A). Furthermore, reperfusion after cardioplegia with HTK solution partially alleviated cardiac injury in comparison to the I/R group. The left ventricular development pressure (LVDP) and left ventricular end diastolic pressure (LVEDP) at the end of reperfusion were significantly improved in the HTK+I/R group (Figure 1B). Notably, there were no significant differences in heart rate among these groups (Figure 1B).

***Melatonin enhanced the cardioprotective effect of HTK solution***

**Figure 2:**
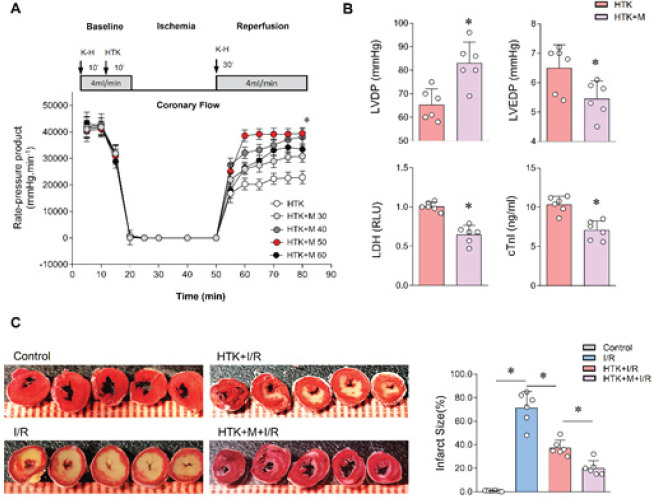
Melatonin improved the protective effects of HTK solution on cardiac function

In order to compare the cardioprotective effects, different concentrations of melatonin (30-60 μmol/l) were added to HTK cardioplegia. The results indicated that melatonin enhanced the protective effect of HTK solution on cardiac systolic and diastolic functions, and reduced the release of cTnl and LDH in the perfusate (Figure 2 A-B). Notably, 50 μmol/L melatonin significantly improved the heart rate systolic pressure product (tension time index, RPP) of isolated perfusion heart. Therefore, this concentration was selected for subsequent experiments.

Then the langendorff-perfused isolated mice hearts from the con, I/R, HTK+I/R, and HTK+M+I/R groups were subjected to TTC staining. It was observed that the myocardial infarct size was reduced in the HTK solution group compared to the I/R group (Figure 2C). Furthermore, the addition of melatonin further decreased the myocardial infarct size after I/R (Figure 2C). These findings indicate that melatonin enhances the protective effects of HTK solution on cardiac function. 

***Melatonin enhanced myocardial energy metabolism of HTK solution***

**Figure 3:**
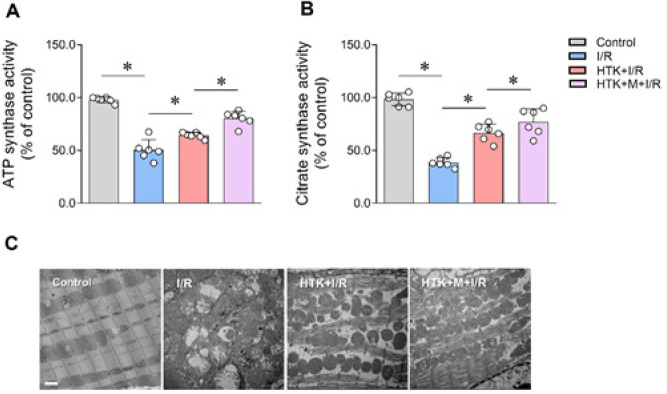
Melatonin improved myocardial energy metabolism of HTK solution

Subsequently, the energy metabolism and mitochondrial status of myocardial tissues were examined in each group. Compared to the control group, the I/R group exhibited a significant decrease in ATP enzyme synthesis activity and citrate synthesis activity (Figure 3 A-B). However, incubation with HTK solution increased myocardial ATP enzyme synthesis activity and citrate synthesis activity (Figure 3 A-B). The addition of melatonin further improved energy metabolism in isolated perfusion heart incubation with HTK solution (Figure 3 A-B). Moreover, electron microscope analysis revealed that melatonin supplementation enhanced the integrity of myocardial mitochondria and mitigated mitochondrial damage compared to the I/R+HTK group (Figure 3C).

***Melatonin ameliorated cardiac ER stress***

**Figure 4:**
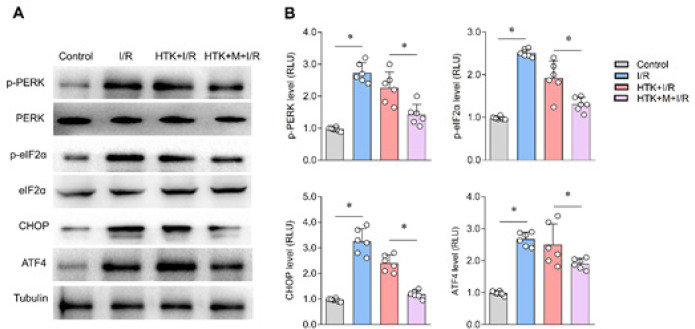
Melatonin ameliorated cardiac ER stress

ER stress plays a crucial role in myocardial I/R injury. In order to investigate this, we examined the expression of ER stress-related proteins in different groups. Our findings revealed that the levels of p-PERK, p-eIF2α, CHOP, and ATF4 were significantly up-regulated in the heart tissue of the I/R group compared to the control group (Figure 4 A-B). Surprisingly, incubation with HTK solution did not reduce their expression. However, the addition of melatonin to the HTK solution resulted in a significant reduction in the phosphorylation of eIF2α, PERK, and CHOP, as well as ATF4 expression, when compared to the HTK+I/R group (Figure 4 A-B). These results indicate that melatonin has an additional cardioprotective effect when combined with HTK solution by inhibiting ER stress.

***Melatonin promoted the inhibition of cardiac carbonyl stress by HTK solution***

**Figure 5:**
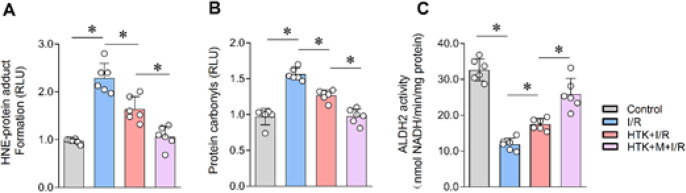
Melatonin promoted the inhibition of cardiac carbonyl stress by HTK solution

Lipid peroxidation and carbonyl stress are also harmful factors that cannot be overlooked in myocardial I/R injury. Previous studies have shown that myocardial carbonyl stress occurs during I/R, characterized by an increase in the content of active carbonyl substance hydroxynonenal (HNE) and a significant rise in protein carbonylation (4, 14, 15). Therefore, we measured the levels of lipid peroxidation and carbonyl stress in heart tissue. As depicted in Figure 5A, HTK solutions reduced cardiac HNE levels after I/R, and the addition of melatonin further decreased HNE levels. Similarly, the same conclusion was reached for the carbonylation content of myocardial proteins (Figure 5B). To evaluate the activity of ALDH2, which is the most important endogenous carbonyl scavenging enzyme responsible for the oxidation of toxic aldehydes to carboxylic acids (14), ALDH2 activity was measured. Interestingly, we found that melatonin further enhanced the HTK solution-induced increase in ALDH2 activity (Figure 5C).

***The enhanced cardioprotective effects of HTK solution were AMPK*** ***α*** ***2 dependent***

**Figure 6:**
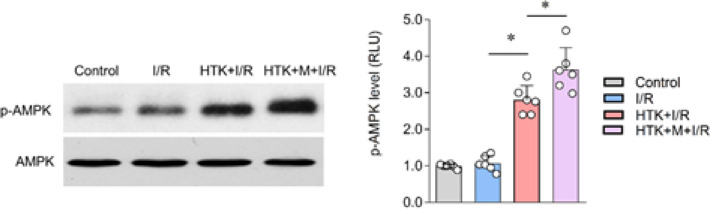
Melatonin promoted the phosphorylation of cardiac AMPK by HTK solution

**Figure 7:**
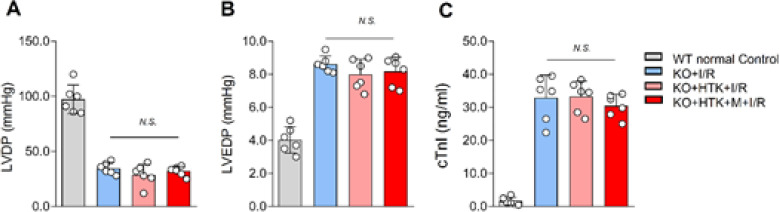
Melatonin failed to improve the cardioprotective effects of HTK solution in AMPKα2 KO mice

To further elucidate the potential mechanism through which melatonin enhanced the cardioprotective effect of HTK solution against I/R injury, the phosphorylation of AMPK was measured. The results showed that the I/R+HTK group effectively increased AMPK phosphorylation in *ex vivo* I/R hearts, and the addition of melatonin to HTK solution further up-regulated AMPK phosphorylation (Figure 6). Subsequently, isolated hearts from AMPKα2 KO mice were perfused in the presence of melatonin and HTK solution, or in the absence of HTK solution alone, to investigate the involvement of AMPKα2 in the protective effect exerted by melatonin. The results indicated that both the I/R+HTK and I/R+M+HTK groups were unable to reduce the myocardial infarction size, and there was no improvement in LVDP and LVEDP, as well as decrease in cTnl release (Figure 7 A-C). These findings suggest that melatonin mediates AMPKα2 to enhance the myocardial protective effect of HTK solution. 

## Discussion

In recent years, there have been numerous reports on the significant role of melatonin in inhibiting various pathophysiological processes associated with cardiovascular diseases (9, 16, 17). A perfusion study conducted on isolated mouse hearts revealed that melatonin effectively suppressed the opening of mitochondrial permeability transition pores, reduced lipid oxidation levels, and prevented the release of mitochondrial cytochrome C, thereby leading to a significant reduction in myocardial infarction area (18). Similarly, in a rat model of myocardial I/R injury, melatonin demonstrated the ability to maintain mitochondrial structure stability in ischemic cardiomyocytes, inhibit cardiomyocyte apoptosis, and enhance ATP synthesis, ultimately improving cardiac function (19). Preconditioning with melatonin prior to cardiac I/R injury has also shown promising results, including reduced peroxidation injury, inhibition of cardiomyocyte apoptosis, and improved cardiac function (20). Furthermore, studies have indicated that melatonin protects against myocardial I/R injury by activating the SIRT1 pathway mediated by melatonin receptors. Our previous studies have confirmed that melatonin reduces myocardial ischemic vulnerability caused by chronic pain by inhibiting RIP3-MLKL/CaMKII-dependent programmed necrosis (21). These studies collectively suggest that melatonin plays a significant role in reducing myocardial I/R injury. While numerous articles have reported the positive properties of melatonin, such as reducing apoptosis, protecting mitochondria, promoting ATP production, reducing lactic acid concentration, and improving Ca^2+^ regulation. 

As shown by Figure 2, we observed the concentration gradient of myocardial protection induced by melatonin in HTK solution. The results showed that 50 μM melatonin had the best protective effect, and the protective effect was better than 60 μM, which significantly improved the product of heart rate and systolic blood pressure and decreased LDH and cTnl. This concentration is also consistent with the previous reports, which was also confirmed by the studies of Kaneko, Lochner, Genade, and others (22-24). In addition, existing studies have shown that the concentration range of cellular protection of melatonin is relatively broad because of its multiple cytoprotective effects, such as free radical scavenging and broad-spectrum antioxidant properties (23), the intracellular regulation of calcium (25), and promoting survival (26).

We have the same aim as Schaefer *et al*. to study whether HTK in cardioplegic solution can be improved by melatonin, but different types of animal models and ischemic methods are used. Previous studies have also shown that the cardioprotective efficiency of HTK or HTK-N depends on the type of ischemia (27). However, they focused on the fact that melatonin as a ROS scavenger could reduce the outbreak of ROS after ischemia after adding the HTK solution, but the results showed that tissue damage of ROS seemed to be less important for the selected ischemic condition, because melatonin did not improve the functional recovery of the heart protected by HTK during reperfusion. In other words, they found that melatonin was ineffective in improving heart protection without or with HTK, specifically, melatonin did not enhance the poorer functional parameters of HTK-protected hearts compared to untreated hearts (25). 

Schaefer *et al*. found that melatonin had no effect on diastolic blood pressure during reperfusion, but melatonin during reperfusion had a significant negative effect on left ventricular pressure (25). Not only that, another study also found that melatonin has a cardioprotective effect in rat hearts, but the contractile force is also severely limited (28). However, in our experiment, we found that left ventricular developed pressure (LVDP) and left ventricular end-diastolic pressure (LVEDP) at the end of reperfusion in the HTK+I/R group were significantly better than those in the Imax R group. Moreover, the addition of melatonin further enhanced the myocardial protective effect of HTK: compared with the simple HTK group, increased LVDP after reperfusion, decreased LVEDP, and significantly reduced the release of LDH and cTnl in perfusate. In Schaefer *et al*. experiments, at the end of reperfusion after 80 min of warm ischemia at 30 ^°^C, the ventricular function of the untreated hearts was better than that of the HTK-treated hearts (25). The results showed that reperfusion times of less than 3 hr (2 hr for crystalloid-perfused Langendorff hearts) are unreliable with this method since insufficient washout time has occurred (29). However, in our experiment, we detected that HTK+melatonin was given before ischemia, and the isolated whole heart was subjected to ischemia for 30 min and then reperfusion for 120 min. So we used TTC staining to observe myocardial infarction after reperfusion, and the results showed that there was obvious myocardial infarction in the ischemia-reperfusion group. Compared with the ischemia-reperfusion group, the myocardial infarct size of the HTK group decreased. The addition of melatonin can further reduce the myocardial infarct size and improve the myocardial protective effect.

The initial factor of ischemia-reperfusion injury is the disturbance of myocardial energy metabolism. We then try to explore whether melatonin can enhance the myocardial protective function of HTK solution by inhibiting mitochondrial damage and energy metabolism disorder caused by ROS and calcium overload. Our study found that the ATP enzyme synthesis activity and citrate synthesis activity of mice decreased significantly during ischemia-reperfusion injury, while the enzyme activity of mice treated with HTK cardioprotective solution increased significantly, and the ATP enzyme activity and citrate enzyme activity were further restored after the addition of melatonin. This suggests that the addition of melatonin can enhance the protective effect of HTK cardioprotective solution on the energy metabolism of cardiomyocytes during ischemia-reperfusion injury. After perfusion *in vitro*, the sections of the mouse heart were removed and fixed, and it was observed by the electron microscope that the mitochondria of mouse cardiomyocytes were swelling, exhibiting crests, rupturing, and developing vacuoles after ischemia-reperfusion. However, the injury of mitochondria was significantly reduced by the addition of HTK cardioprotective solution, and further alleviated by the addition of melatonin. These results suggest that the increase of melatonin can effectively enhance the protective effect of HTK myocardial protective solution on mitochondria during ischemia-reperfusion.

The occurrence of ischemia, ATP depletion, production of free radicals, and disruption of Ca^2+^ homeostasis can lead to ER dysfunction and trigger ER stress. However, if ER stress persists or becomes excessive, it can disrupt Ca^2+^ homeostasis and induce apoptosis, resulting in tissue injury. This process plays a crucial role in the development of myocardial I/R injury (30-33). Therefore, we examined the levels of ER stress-related proteins in isolated mouse hearts during simulated myocardial I/R. PERK, eIF2α, CHOP, and ATF4 are marker proteins of ER stress. Our study revealed that the HTK solution was unable to inhibit ER stress, but the addition of melatonin effectively reduced ER stress.

Numerous studies have demonstrated that activation of AMPK can mitigate the injury to central myocytes during I/R by inhibiting ER stress, and apoptosis, and by regulating energy metabolism and autophagy (34-41). In a healthy myocardium, AMPK is typically activated when mitochondrial function is impaired (e.g., berberine alters ATP/ADP ratio and activates AMPK by inhibiting mitochondrial function). However, this approach does not apply to patients undergoing cardiopulmonary bypass (CPB). Inhibiting mitochondrial function may reduce the oxygen demand, but it is not feasible for CPB patients who already have a compromised energy supply. Therefore, finding an effective method to activate AMPK in patients undergoing CPB cardiac surgery is crucial for regulating glucose metabolism, maintaining mitochondrial activity, providing myocardial energy, and reducing mitochondrial oxidative stress damage. Further research on CPB cardioprotection should focus on addressing this key issue. Therefore, the purpose of this study was to explore the relationship between the protective effects of melatonin-enhanced HTK solution on I/R injury and AMPK. Our results demonstrate that the enhancement of myocardial protective effects of melatonin on HTK solution is closely associated with myocardial AMPKα2.

As early as 2000, a study found that 5-methoxy-N-acetyl-tryptamine, melatonin, and Smethoxy-carbonylamino-N-acetyl-tryptamine, a structurally related indole compound, both protect against myocardial ischemia-reperfusion injury in the isolated rat heart (42). As free radicals play a pivotal role in I/R injury in the past, it was speculated that melatonin induced its cardioprotective power mainly through its free radical scavenging activities and the direct activation of antioxidant enzymes. However, in 2006, researchers (23)showed that infarct size reduction by melatonin was completely abrogated by coadministration of the melatonin receptor antagonist luzindole, whereas luzindole possesses antioxidant qualities itself (43). Thus, it seems that also the melatonin receptors play a critical role in melatonin-induced cardioprotection. After that, Stroethoff *et al*. used the isolated heart and placed it on the Langendorff system and combined ramelteon with luzindole, a melatonin receptor antagonist, and determined the reduction of ischemia and reperfusion injury by ramelteon is mediated by melatonin receptor (44). The action mechanism of melatonin in HTK solution is worthy of further study.

This study primarily focused on improving HTK myocardial protective solution, which is a necessary means of protection in cardiac surgery. The use of melatonin alone is insufficient to effectively maintain heart function; therefore, this experiment did not establish a separate experimental group using melatonin alone.

A-B) In Langendorff-perfused isolated mice hearts from con, I/R, and HTK+I/R, LVP (A), LVDP, LVEDP, and HR (B) were recorded

Data were presented as mean±SEM, n=6 mice per group. *P<0.05 vs control group, ^# ^*P<*0.05 vs I/R group

A) Hemodynamics of the Langendorff-perfused isolated mice hearts of HTK and HTK+M mice. B) LVDP, LVEDP, and cardiac injury markers were measured. Data were presented as mean±SEM, n=6 mice per group. *P<0.05 vs control group, #*P<*0.05 vs I/R group. C) The Langendorff-perfused isolated mice hearts from con, I/R, HTK+I/R, and HTK+M+I/R groups were used for TTC staining to measure cardiac infarct size, representative images (left) and quantitative analysis of infarct size (right) were shown. Data were presented as mean±SEM, n=6 mice per group. *P<0.05

A) ATP synthase activity and B) citrate synthase activity were detected. Data were presented as mean±SEM, n=6 mice per group. *P<0.05. C) Representative transmission electron microscopic images of isolated perfused mouse hearts from con, I/R, HTK+I/R, and HTK+M+I/R groups. n=6 mice per group

A) Representative western blot data for cardiac p-PERK, p-eIF2α, PERK, eIF2α, CHOP, and ATF4 expression in hearts from control, I/R, HTK, and HTK+M. B) Quantitative analysis of panel, Figure A. Data were presented as mean±SEM, n=6 mice per group. *P<0.05 vs Control group, *P<0.05 vs I/R group

Quantitative analysis of A) 4-HNE, B) protein carbonyl, and C) ALDH2 activity in isolate perfused mice hearts

Data were presented as mean±SEM, n=6 mice per group. *P<0.05 vs Control group, *P<0.05 vs I/R group

Representative Western blots data and quantitative analysis of p-AMPK level

Data were presented as mean±SEM, n=6 mice per group. *P<0.05 vs Control group, *P<0.05 vs I/R group

A and B) Hemodynamics of the Langendorff-perfused isolated hearts from con, I/R, HTK, and HTK+M in AMPKα2 KO mice. C) quantitative analysis of coronary effluent cTnl. Data were presented as mean±SEM, n=6 mice per group

## Conclusion

In this study, we investigated the impact of melatonin on the protective effects of HTK solution in Langendorff-perfused mouse hearts that underwent I/R. Our results showed that melatonin improved energy metabolism and cardiac function, reduced mitochondrial damage and cardiac infarct size, and inhibited cardiac carbonyl stress. Interestingly, while the HTK solution alone did not inhibit ER stress, the addition of melatonin effectively reduced ER stress. Furthermore, we found that the myocardial protective effects of melatonin on HTK solution were closely associated with myocardial AMPKα2. In conclusion, our study suggests that the strategy aimed at enhancing the cardioprotective effects of HTK solution is feasible and may have potential applications in protecting other organs from injury following organ transplantation.

Combined with the literature, we believe that myocardial ischemia-reperfusion injury is an important factor restricting cardiac function after operation, which is characterized by disturbance of myocardial energy metabolism, injury caused by free radicals, and intracellular calcium overload. A series of processes that can lead to cardiomyocyte injury, such as autophagy dysfunction, mitochondrial injury, endoplasmic reticulum stress, carbonyl stress, and so on, will appear in myocardial ischemia-reperfusion injury. The use of small molecular drugs for exogenous treatment or the activation of an endogenous protection mechanism for the prevention and treatment of myocardial ischemia-reperfusion injury are the two mainstream research directions. Although the current basic research on endogenous and exogenous myocardial ischemia protection points out that ischemia-reperfusion injury can be inhibited by inhibiting the above adverse reactions, and also reveals the related mechanism of myocardial protection, the potential mechanism of prevention and treatment of myocardial ischemia-reperfusion injury is far from elucidated, how to use drug combination therapy to inhibit the pathophysiological process of myocardial ischemia-reperfusion injury through a variety of protective ways. In order to effectively protect myocardial function and improve the prognosis of patients will become an important research topic. This study focused on myocardial ischemia-reperfusion injury, carried out some research work *in vitro*, and provided an experimental basis for the prevention and treatment of myocardial ischemia-reperfusion injury.

## Authors’ Contributions

W YS, M H, and Z ZH designed the experiments; S MC and W YS performed experiments and collected data; S MC, Z ZH, Y Y, J WH, ZC, G CH, W YS, and M H discussed the results and strategy; M H, W YS, and G CH supervised, directed, and managed the study; S MC, Z ZH, Y Y, J WH, Z C, G CH, W YS, and M H approved the final version to be published.

## Institutional Review Board Statement

The animal study protocol was approved by the Ethics Committee of Fourth Military Medical University, China.

## Data Availability Statement

The data that support the findings of this study are available upon request from the corresponding authors.

## Conflicts of Interest

The authors declare no conflicts of interest.
